# Association of sugar-sweetened beverage consumption with psychological symptoms among Chinese university students during the COVID-19 pandemic

**DOI:** 10.3389/fpsyg.2022.1024946

**Published:** 2022-10-13

**Authors:** Yujie Wang, Cunjian Bi, He Liu, Hongniu Lin, Ruibao Cai, Jie Zhang

**Affiliations:** ^1^Public Teaching Department, Zhumadian Vocational and Technical College, Zhumadian, China; ^2^School of Physical Education, Chizhou University, Chizhou, China; ^3^Sports Health Promotion Center, Chizhou University, Chizhou, China; ^4^Research Department of Physical Education, Xinjiang University, Urumqi, China

**Keywords:** SSB consumption, psychological symptoms, college students, association analysis, COVID-19

## Abstract

**Background:**

Mental health of college students has become a public health issue of common concern worldwide. Especially during the COVID-19 pandemic, the problem has become even more acute. The aim of this study was to assess the association between sugar-sweetened beverages (SSB) consumption and psychological symptoms among Chinese college students in order to promote their mental health.

**Methods:**

The study population was 6,120 college students aged 19–22 years from Anhui, Henan and Xinjiang, China. Basic demographic information, covariates and SSB Consumption data were collected through a self-assessment questionnaire. The “Multidimensional Sub-health Questionnaire of Adolescents” was used to assess the psychological symptoms of college students. The Chi-square test, one-way ANOVA and logistic regression analysis were used to investigate the differences and associations between SSB consumption and psychological symptoms among Chinese college students.

**Results:**

The detection rate of psychological symptoms among Chinese college students was 8.1%. The detection rate of boys students was 9.4% and that of girls students was 7.1%, and the difference was statistically significant in comparison (*χ*^2^-value was 11.08, *p* < 0.001). After controlling for covariates, Model 2 analysis showed that compared to SSB consumption <2 time/week, college students with SSB consumption ≥2 time/week (*OR* = 2.96, 95% *CI*: 2.36, 3.70) had a higher risk of psychological symptoms (*p* < 0.001). The same trend was found for emotional symptoms, behavioral symptoms, and social adaptation difficulties dimensions.

**Conclusion:**

There is an association between SSB consumption and the occurrence of psychological symptoms among Chinese college students. Future measures should be taken to reduce both SSB consumption and the incidence of psychological symptoms.

## Introduction

Over the past 2 years or so, the COVID-19 pandemic has become a major public health emergency of international concern all over the world (Chilamakuri and Agarwal, 2021). Evidence suggested that, college students are more vulnerable to sudden changes in COVID-19 pandemic compared to the general population (Stangier et al., 2021). Mental health among college students is a significant public health concern (Mackenzie et al., 2011; Beiter et al., 2015). Several meta-analysis presented that the prevalence of psychological symptoms (e.g., depression, anxiety) among college students greatly increased during the COVID-19 pandemic (Li et al., 2021; Liyanage et al., 2021; Mulyadi et al., 2021; Santabarbara et al., 2021), including in China (Ma et al., 2020; Wang et al., 2021; Wu et al., 2021).

An emerging body of evidence has suggested that diet plays an important role in mental health (Huang et al., 2019). Current evidence also supported that healthy eating patterns that meet food-based dietary recommendations and nutrient requirements may assist in the prevention and treatment of depression and anxiety (Kris-Etherton et al., 2021). Constituting a large part of the energy intake, sugar-sweetened beverages (SSB) has been considered to be an important risk factor for obesity, type 2 diabetes mellitus, cardiovascular disease. The SSB consumptions per person are still increasing (Popkin and Hawkes, 2016). In China, the percentage of SSB consumption in children increased from 72.6 to 90.3% in 2004–2011 (Guo et al., 2021). During the COVID-19 pandemic and lockdown, changes in dietary behavior factors include increased total food consumption, decreased adherence to healthy diets, and increased snack and SSB intake (Chew and Lopez, 2021). It has also been reported that due to emotional As a way of comforting and feeling better in an anxious state, snacking and SSB intake have increased markedly during the COVID-19 pandemic (Di Renzo et al., 2020; Scarmozzino and Visioli, 2020).

A meta-analysis including 10 observational studies involving 365,289 participants indicated that SSB consumption might be associated with a modestly higher risk of depression (Hu et al., 2019). It was reported that consuming SSB 1 or more times per day versus consuming none was associated with a 26% greater prevalence of poor mental health (95% *CI*: 1.11–1.43) among US adults (Freije et al., 2021). Similar results were also found in Korean (Kim et al., 2022), Canada (Yu et al., 2017) and Chinese (Yu et al., 2015) adults and children and adolescents from Iranian (Zahedi et al., 2014) and China (Liu et al., 2022). A prospective data suggested that high consumption of SSB during adolescence were associated with psychological distress in young adulthood (Kleppang et al., 2021). A study of Tibetan adolescents at high altitudes in China showed an association between SSB consumption and executive function (Zhang et al., 2022). A study of Australian women during the COVID-19 pandemic showed a strong positive association between SSB consumption days and psychological distress, and SSB consumption days were higher during the COVID-19 pandemic (Grieger et al., 2022). However, there are few such studies on Chinese college students.

There is evidence to show that, 9 months after initiation of the outbreak of COVID-19, More than half of Chinese college students have depressive and anxiety symptoms (Xiao et al., 2021). In contrast, a large proportion of college students consumed SSB (82.0, 95%CI, 81.4, 82.6; Rahman et al., 2022). This study hypothesized that there is an association between SSB consumption and psychological symptoms of Chinese college students. Our study focuses on exploring whether there is an association between SSB consumption and psychological symptoms among Chinese college students during the COVID-19 pandemic. To provide reference for Chinese college students to control SSB consumption and reduce the incidence of psychological symptoms.

## Materials and methods

### Study design and population

**Figure 1:**
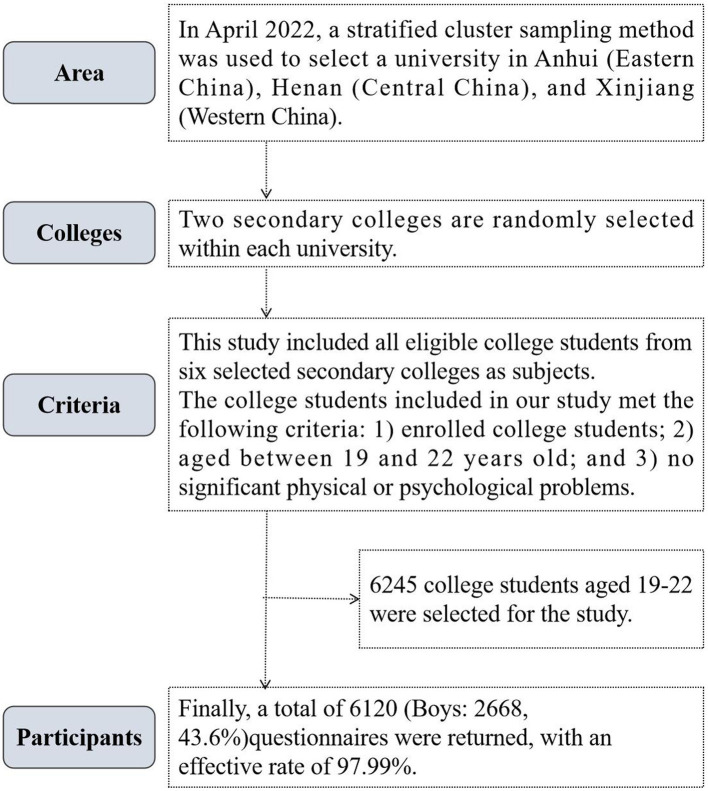
Sampling process of Chinese college students’ subjects.

Our study was a cross-sectional original study. In April 2022, a stratified whole-group sampling method was used to sample subjects in two steps. First, one university in Anhui (Eastern China), one in Henan (Central China) and one in Xinjiang (Western China) were selected. Second, two colleges were randomly selected from each school through communication with the school administration. All eligible students participated in the survey. The college students included in our study met the following criteria: (1) enrolled college students; (2) aged between 19 and 22 years old; (3) by asking the head teacher, the included subjects had no significant physical or psychological problems. Finally, 6,245 college students aged 19–22 were selected for the study, and 6,120 (boys: 2668, 43.6%) questionnaires were returned, with an effective rate of 97.99%. The specific sampling process of the subjects is shown in Figure 1.

The written informed consent of the student was obtained prior to the survey, which was conducted anonymously and encoded. This research survey was approved by the Human Ethics Committee of Chizhou University (202201051).

#### Procedure

Under the cooperation of teachers and accompanied by graduate students majoring in human movement science, college students completed the questionnaire independently. An electronic questionnaire containing basic information, sugary drinks, and psychological symptoms was distributed to the students in the classroom. Students obtained the electronic questionnaire by scanning a QR code and submitted it on the spot when finished.

#### SSB consumption

Data on SSB consumption were obtained using the following question: In the past 7 days, how many time did you drink SSB. SSB include milk tea, carbonated beverages, non-carbonated sugar-sweetened non-alcoholic beverages, sports beverages, fruit juice beverages, sweetened milk and yogurt beverages, vitamin water, and sweetened iced tea that are common in our daily lives, such as Natural fruit juices, Coke, Sprite, Nutrition Express, Red Bull etc.?” (0, 1, 2, 3, 4, ≥5)? About 250 ml is consumed each time. To ensure an adequate number of participants in each group, the frequency of SSB consumption was categorized into two groups (<2 time/week, and ≥2 times/week).

#### Psychological symptoms

The Multidimensional Sub-health Questionnaire of Adolescents (MSQA) is a psychological symptoms designed specifically for a group of children and adolescents (including college students) and has been used in several studies (Yao et al., 2015; Yang et al., 2019; Mahara et al., 2021; Huang et al., 2022). In this paper, the survey of psychological symptoms was completed using a simple version of the MSQA with good reliability and validity. The short version of the questionnaire contains 15 items to investigate emotional symptoms (7 items, e.g., “often blaming yourself”), behavioral symptoms (4 items, e.g., “always feeling like people are working against you”), and social adaptation difficulties (4 items, e.g., “unwilling to ask for help when in trouble”). There are six response options for each items: “more than 3 months,” “more than 2 months,” “more than 1 month,” “more than 2 weeks,” “more than 1 week,” “never or less than 1 week.” Participants choose one option that can best describe their situations. The score is recorded as 1 if the response was “more than 1 month,” “more than 2 months,” and “more than 3 months” and recorded as 0 for the other responses. Scores for the three dimensions (emotional symptoms, behavioral symptoms, and social adaptation difficulties) were obtained by summing the scores for the corresponding items. Psychological symptoms were calculated by summing the scores of the all the 15 items. The cutoff points for emotional symptoms, behavioral symptoms, and social adaptation difficulties were ≥4, ≥1, and ≥2, respectively; the cutoff for psychological symptoms was ≥7.

#### Covariates

Covariates investigated in our study included siblings, parental education, body mass index (BMI), Socioeconomic Status (SES), sleep duration, screen time and Moderate and Vigorous Physical Activity (MVPA). Parental education is divided into primary school and below, junior high school and high school, college and above. BMI is calculated as weight (kg)/height (m)^2^. The SES survey includes surveys of parents’ occupations, educational attainment, and household facilities, and a composite score is calculated. According to the final score of the survey, SES defines the low grade according to the percentile <15th percentile, 15–85th percentile as the middle grade, and >85th percentile as the high grade. Sleep duration is divided into ≥8 h/days, <8 h/days. Screen time is divided into <2 h/days, ≥2 h/days. MVPA was calculated based on the time and frequency of moderate-to-high-intensity exercise that participants filled out each day for the past week. MVPA is defined as an activity performed with moderate or high effort while exercising, and one feels short of breath. Such as cycling, brisk running, playing football, lifting heavy objects, skating, etc.

#### Statistical analysis

Descriptive language was used to express the SSB status of different categories of Chinese college students. The description of continuous variables was expressed as mean ± standard deviation (M ± SD). Categorical variables are expressed as percentages (%). The detection rate of psychological symptoms and its three dimensions for Chinese college students with different SSB was analyzed by Chi-squares test. The relationship between SSB consumption and psychological symptoms and its three dimensions was analyzed by logistic regression using three models (Crude Model, Model 1, Model 2). Crude Model was conducted without adjustment; Model 1 was conducted after adjusting age, siblings, parental education, BMI, SES; Based on Model 1, Model 2 included sleep duration, screen time and MVPA as additional control variables. *α* = 0.05 was used as the two-sided test level.

## Results

We conducted the SSB consumption survey on 6,120 (boys: 2668, 43.6%) Chinese college students, with an average age of (20.16 ± 1.03) years. In China, 12.2% (748/6120) of college students had SSB consumption ≥2 time/week.

The results showed that the detection rate of psychological symptoms among Chinese college students was 8.1% (495/6120). The detection rate of psychological symptoms problems was 9.4% (251/2668) in boys students and 7.1% (244/3452) in girls students, and the difference was statistically significant in comparison (*χ*^2^-value of 11.08, *p* < 0.01).

Overall, among college students with SSB consumption <2 time/week and SSB consumption ≥2 time/week, the detection rate of psychological symptoms was similar in terms of sex, only child, father’s education, mother’s education, sleep duration, screen time, and socioeconomic status, the differences were statistically significant. The detection rate of psychological symptoms of college students with SSB consumption ≥2 time/week was higher than that of college students with SSB consumption<2 time/week (Table 1).

**Table 1:** Comparison of SSB consumption status of college students with different characteristics in China.

Characteristics	Psychological symptoms			*χ* ^2^-value	*p*-value
	Total	SSB consumption <2 time/week	SSB consumption ≥2 time/week		
*Sex*					
Boys	251 (9.4)	179 (7.8)	72 (19.5)	50.925	<0.001
Girls	244 (7.1)	165 (5.4)	79 (20.9)	123.620	<0.001
*Only child*					
Yes	90 (6.1)	43 (3.4)	47 (22.4)	113.961	<0.001
No	405 (8.7)	301 (7.3)	104 (19.3)	85.825	<0.001
*Father’s education*					
Primary school and below	207 (11.8)	143 (9.9)	64 (20.9)	29.522	<0.001
Junior high school and above	288 (6.6)	201 (5.1)	87 (19.7)	136.759	<0.001
*Mother’s education*					
Primary school and below	166 (6.1)	104 (4.5)	62 (16.4)	80.092	<0.001
Junior high school and above	329 (9.6)	240 (7.9)	89 (24.1)	99.419	<0.001
*Sleep duration*					
≥8 h/days	40 (2.4)	34 (2.2)	6 (5.2)	4.319	0.038
<8 h/days	455 (10.3)	310 (8.2)	145 (22.9)	128.153	<0.001
*Screen time*					
<2 h/days	53 (3.8)	44 (3.4)	9 (9.6)	9.329	0.002
≥2 h/days	442 (9.4)	300 (7.4)	142 (21.7)	136.128	<0.001
*Socioeconomic status (SES)*					
Low	97 (10.1)	81 (9.8)	16 (11.7)	0.455	0.500
Medium	305 (7.1)	210 (5.5)	95 (19.5)	129.131	<0.001
High	93 (11.0)	53 (7.4)	40 (32.3)	67.013	<0.001

Descriptive statistics are presented as mean (standard deviation) and number (percentage) for continuous and categorical. SSB consumption is calculated in 250 ml bottles for each calculation. SES, low (<15th percentile), medium (15–85th percentile), high (>85th percentile). SES, socioeconomic status; BMI, body mass index; MVPA, moderate and vigorous physical activity; SSB, sugar-sweetened beverages.

Overall, the detection rate of psychological symptoms was 20.2% among college students with SSB consumption ≥2 time/week, which was higher than that of those with SSB consumption <2 time/week (6.4%). Regarding the different dimensions, emotional symptoms, behavioral symptoms, and social adaptation difficulties were detected at the highest rates among college students with SSB consumption ≥2 time/week, 26.3, 30.6, and 26.5% respectively, the differences were statistically significant (*χ*^2^-value was 302.786, 354.593, 272.343, respectively; *p* < 0.001; Table 2).

**Table 2:** Comparison of the detection rate of psychological symptoms among college students with different SSB consumption in China.

Psychological symptoms	SSB Consumption	No psychological symptoms		Have psychological symptoms		*χ* ^2^-value	*p*-value
		*N*	Percentage	*N*	Percentage		
*Boys*							
Emotional symptoms	<2 time/week	2,117	92.1	181	7.9	75.733	<0.001
	≥2 time/week	287	77.6	83	22.4		
Behavioral symptoms	<2 time/week	2091	91.0	207	9.0	127.664	<0.001
	≥2 time/week	261	70.5	109	29.5		
Social adaptation difficulties	<2 time/week	2,122	92.3	176	7.7	69.120	<0.001
	≥2 time/week	291	78.6	79	21.4		
Psychological symptoms	<2 time/week	2,119	92.2	179	7.8	50.925	<0.001
	≥2 time/week	298	80.5	72	19.5		
*Girls*							
Emotional symptoms	<2 time/week	2,892	94.1	182	5.9	252.252	<0.001
	≥2 time/week	264	69.8	114	30.2		
Behavioral symptoms	<2 time/week	2,856	92.9	218	7.1	231.641	<0.001
	≥2 time/week	258	68.3	120	31.7		
Social adaptation difficulties	<2 time/week	2,853	92.8	221	7.2	223.707	<0.001
	≥2 time/week	259	68.5	119	31.5		
Psychological symptoms	<2 time/week	2,909	94.6	165	5.4	123.620	<0.001
	≥2 time/week	299	79.1	79	20.9		
*Total*							
Emotional symptoms	<2 time/week	5,009	93.2	363	6.8	302.786	<0.001
	≥2 time/week	551	73.7	197	26.3		
Behavioral symptoms	<2 time/week	4,947	92.1	425	7.9	354.593	<0.001
	≥2 time/week	519	69.4	229	30.6		
Social adaptation difficulties	<2 time/week	4,975	92.6	397	7.4	272.343	<0.001
	≥2 time/week	550	73.5	198	26.5		
Psychological symptoms	<2 time/week	5,028	93.6	344	6.4	167.798	<0.001
	≥2 time/week	597	79.8	151	20.2		

After controlling for covariates, Model 2 analysis showed that compared to SSB consumption <2 time/week, college students with SSB consumption ≥2 time/week (*OR* = 2.96, 95% *CI*: 2.36, 3.70) had a higher risk of psychological symptoms (*p* < 0.001). The same trend was found for emotional symptoms, behavioral symptoms, and social adaptation difficulties dimensions. And the trends were consistent for college boys and girls (Table 3).

**Table 3:** Logistic regression analysis of SSB consumption and psychological symptoms among Chinese college students (*n* = 6,120).

Psychological symptoms	SSB Consumption	Odds ratio (95% confidence interval)		
		Crude Model	Model 1	Model 2
*Boys*				
Emotional symptoms	<2 time/week	1.00 (Reference)	1.00 (Reference)	1.00 (Reference)
	≥2 time/week	3.38 (2.54, 4.51)^a^	2.82 (2.05, 3.88)^a^	2.82 (2.05, 3.89)^a^
	*p* for trend	<0.001	<0.001	<0.001
Behavioral symptoms	<2 time/week	1.00 (Reference)	1.00 (Reference)	1.00 (Reference)
	≥2 time/week	4.22 (3.24, 5.50)^a^	3.94 (2.96, 5.25)^a^	3.61 (2.70, 4.83)^a^
	*p* for trend	<0.001	<0.001	<0.001
Social adaptation difficulties	<2 time/week	1.00 (Reference)	1.00 (Reference)	1.00 (Reference)
	≥2 time/week	3.27 (2.44, 4.39)^a^	2.92 (2.12,4.01)^a^	2.86 (2.07,3.96)^a^
	*p* for trend	<0.001	<0.001	<0.001
Psychological symptoms	<2 time/week	1.00 (Reference)	1.00 (Reference)	1.00 (Reference)
	≥2 time/week	2.86 (2.12,3.86)^a^	2.20 (1.59,3.05)^a^	1.90 (1.37,2.64)^a^
	*p* for trend	<0.001	<0.001	<0.001
*Girls*				
Emotional symptoms	<2 time/week	1.00 (Reference)	1.00 (Reference)	1.00 (Reference)
	≥2 time/week	6.86 (5.26,8.95)^a^	8.06 (6.00,10.83)^a^	6.98 (5.18,9.40)^a^
	*p* for trend	<0.001	<0.001	<0.001
Behavioral symptoms	<2 time/week	1.00 (Reference)	1.00 (Reference)	1.00 (Reference)
	≥2 time/week	6.09 (4.71, 7.88)^a^	6.83 (5.17, 9.02)^a^	5.92 (4.47, 7.85)^a^
	*p* for trend	<0.001	<0.001	<0.001
Social adaptation difficulties	<2 time/week	1.00 (Reference)	1.00 (Reference)	1.00 (Reference)
	≥2 time/week	5.93 (4.59, 7.67)^a^	7.42 (5.58, 9.85)^a^	6.44 (4.83, 8.58)^a^
	*p* for trend	<0.001	<0.001	<0.001
Psychological symptoms	<2 time/week	1.00 (Reference)	1.00 (Reference)	1.00 (Reference)
	≥2 time/week	4.66 (3.47, 6.25)^a^	5.49 (3.98, 7.56)^a^	4.70(3.40,6.50)^a^
	*p* for trend	<0.001	<0.001	<0.001
*Total*				
Emotional symptoms	<2 time/week	1.00 (Reference)	1.00 (Reference)	1.00(Reference)
	≥2 time/week	4.93 (4.06,5.99)^a^	4.62 (3.75,5.69)^a^	4.31 (3.49,5.31)^a^
	*p* for trend	<0.001	<0.001	<0.001
Behavioral symptoms	<2 time/week	1.00 (Reference)	1.00 (Reference)	1.00 (Reference)
	≥2 time/week	5.14 (4.27,6.18)^a^	5.08 (4.18,6.17)^a^	4.55(3.73,5.54)^a^
	*p* for trend	<0.001	<0.001	<0.001
Social adaptation difficulties	<2 time/week	1.00 (Reference)	1.00 (Reference)	1.00 (Reference)
	≥2 time/week	4.51 (3.72,5.47)^a^	4.36 (3.57, 5.34)^a^	3.99 (3.26, 4.91)^a^
	*p* for trend	<0.001	<0.001	<0.001
Psychological symptoms	<2 time/week	1.00 (Reference)	1.00 (Reference)	1.00 (Reference)
	≥2 time/week	3.70 (3.00, 4.56)^a^	3.42 (2.74, 4.27)^a^	2.96 (2.36, 3.70)^a^
	*p* for trend	<0.001	<0.001	<0.001

Model 1 controlled for age, only child, parental education, BMI, and SES; Model 2 controlled for Sleep duration, screen time, and MVPA on top of Model 1. SES, socioeconomic status; BMI, body mass index; MVPA, moderate and vigorous physical activity; SSB, sugar-sweetened beverages.

a *p* < 0.001.

## Discussion

Our study investigated SSB consumption and psychological symptoms of Chinese college students, and analyzed the association between the two. Our results showed a strong association between SSB consumption and psychological symptoms among Chinese college students. After controlling for relevant demographic factors and confounders, compared to SSB consumption <2 time/week, college students with SSB consumption ≥2 time/week had a higher risk of psychological symptoms.

More and more studies have confirmed that SSB consumption among children and adolescents is increasing rapidly year by year, and college students are an important stage of SSB consumption and an important group of SSB consumption (Sharma et al., 2020). The Scientific Research Report on Dietary Guidelines for Chinese Residents (2021) shows that the sales of sugary beverages are increasing year by year, and children and adolescents have the highest consumption rate of sugary beverages at more than 30 and 25%, which should draw sufficient attention (Residents, 2021). In addition, 42.1% of free sugar intake in urban population comes from sugary drinks and dairy beverages. Studies have confirmed that excessive SSB consumption will have more adverse effects on the physical and mental health of children and adolescents (Russo et al., 2020; Roesler et al., 2021). Studies have shown that during the COVID-19 pandemic lockdown, lifestyle changes such as decreased physical activity and increased SSB consumption have led to increased obesity (Sanchez et al., 2021; Pulvera et al., 2022). The occurrence of obesity due to excessive consumption of SSB is also an important factor to further increase the occurrence of psychological symptoms, which has adverse effects on mental health. This is an important reason for the higher detection rate of psychological symptoms in college students with higher SSB consumption.

Previous studies have tended to analyze the relationship between SSB consumption and physical function, while relatively few studies have addressed the relationship between SSB consumption and mental health, especially in college student populations with high and unstable psychological burdens. Our study analyzed the association between SSB consumption and psychological symptoms among Chinese college students. The results showed the association between SSB consumption and psychological symptoms. Our research also showed that the detection rate of psychological symptoms was significantly higher among Chinese college boys (9.4%) than girls (7.1%), which is consistent with the findings of related studies (Sagar-Ouriaghli et al., 2020).One of the main reasons is that, influenced by the traditional Chinese educational ideology, families and society give college boys greater hopes and responsibilities, which invariably increases the psychological burden of college boys, resulting in higher psychological symptoms than girls. Secondly, compared with girls students, boys college students have more bad life and eating habits, such as smoking, alcoholism, staying up late, drinking too many sugary drinks, obesity, etc. These bad life or eating habits are risk factors for the occurrence of psychological symptoms problems.

There are also some research explanations on the reciprocal mechanism of SSB consumption and psychological symptoms. Studies have confirmed that excessive intake of SSB in children and adolescents promotes the secretion of dopamine in the brain, which makes the body feel pleasure and stimulates the increase of appetite, leading to the occurrence of overweight and obesity, thus leading to the occurrence of psychological symptoms (Anjum et al., 2018). It was also confirmed that high sugar consumption groups (>100 g/days) were significantly associated with higher fatigue, social activity distress, and depression compared to low sugar consumption groups (<100 g/days) (Maaz et al., 2020). Potential deleterious effects of low to moderate intake of SSBs on cardiovascular risk markers such as low-density lipoprotein particles, fasting glucose and high sucrose C-reactive protein that contribute to an increased risk of psychological symptoms (Aeberli et al., 2011). In addition to fructose intake that promotes insulin resistance, hypertension, and dyslipidemia, leading to metabolic dysregulation, fructose intake stimulates the hypothalamic–pituitary–adrenal (HPA) axis, leading to elevated glucocorticoids, which in turn Elevated hormones are often associated with serious psychiatric consequences, such as suicide, psychotic disorders, depression, mania, and panic disorder (Harrell et al., 2015).

There are several advantages of this study. First, the study has a larger sample size, which can better represent the situation of SSB consumption and psychological symptoms among Chinese college students, and the results are more convincing. Second, the study is the first to analyze the association between SSB consumption and psychological symptoms among Chinese college students, which provides a reference and help for the development of education and public health policies by Chinese educational and administrative authorities (Farsad-Naeimi et al., 2020; Yin et al., 2021). Our study also has some limitations. For one, this study was a cross-sectional survey study, and it was only able to analyze the association between SSB consumption and psychological symptoms, but not to understand the causal causality between the two. Second, the investigation of covariates in this study was limited, only family factors, parental education, SES, diet, sleep, and physical activity were investigated, and more factors, such as video screen time and academic stress, should be investigated in future studies to better analyze the association that exists between SSB consumption and psychological symptoms. Third, it is difficult to accurately measure SSB consumption due to the influence of the subjects’ recall ability; at the same time, questionnaires assessing mental health may underreport results because students may be ashamed to answer certain questions.

## Conclusion

In conclusion, there was an association between SSB consumption and psychological symptoms among Chinese college students. Future measures should be taken to reduce both SSB consumption and the incidence of psychological symptoms. The results of this study will provide a reference for the primary and secondary prevention of mental health among Chinese university students. It will also provide a reference and help for the development of education and public health policies by Chinese university education and government departments.

## Data availability statement

The raw data supporting the conclusions of this article will be made available by the authors, without undue reservation.

## Ethics statement

The studies involving human participants were reviewed and approved by the Human Ethics Committee of Chizhou University (202201051). This study was conducted in accordance with the Declaration of Helsinki. The patients/participants provided their written informed consent to participate in this study.

## Author contributions

YW and CB: Conceptualization. HL: Data curation. CB: Formal analysis. HL: Funding acquisition. RC: Investigation. JZ: Methodology. YW: Project administration. YW: Resources. CB: Software. RC: Supervision. JZ: Validation. HL: Visualization. YW and CB: Writing – original draft. YW and CB: Writing – review and editing. All authors have read and agreed to the published version of the manuscript.

## Funding

This project is funded by the Anhui Provincial Department of Education “Anhui Province Higher Education Provincial Quality Engineering Major Project“ (2021jyxm1013).

## Conflict of interest

The authors declare that the research was conducted in the absence of any commercial or financial relationships that could be construed as a potential conflict of interest.

## Publisher’s note

All claims expressed in this article are solely those of the authors and do not necessarily represent those of their affiliated organizations, or those of the publisher, the editors and the reviewers. Any product that may be evaluated in this article, or claim that may be made by its manufacturer, is not guaranteed or endorsed by the publisher.
